# High Variability in Pre-Oviposition Time Independent of Diet Available at Eclosion: A key Reproductive Trait in the Ladybird Beetle *Harmonia axyridis* (Coleoptera: Coccinellidae) in Its Native Range

**DOI:** 10.3390/insects12050382

**Published:** 2021-04-25

**Authors:** Séverin Hatt, Naoya Osawa

**Affiliations:** 1 Laboratory of Forest Ecology, Faculty of Agriculture, Kyoto University, Kitashirakawa-Oiwake, Sakyoku, Kyoto 606-8502, Japan; osawa@kais.kyoto-u.ac.jp; 2 Agroecology and Organic Farming Group, University of Bonn, Auf dem Hügel 6, 53121 Bonn, Germany

**Keywords:** predator, fecundity, fitness, starvation, alternative food, *Perilla frutescens*, agroecosystem diversification

## Abstract

**Simple Summary:**

Insect predators need high-quality food to reach sexual maturity and reproduce. Nonetheless, just emerged adults may face starvation when prey are missing, notably in agroecosystems. This study conducted in laboratory conditions explores whether the type of diet (flower food, prey food or no food) available just after adult eclosion is of critical importance for the future fecundity of adult ladybird beetles. Diet readily available just after emergence did not affect the fecundity and egg viability of *Harmonia axyridis*. Time from eclosion to first egg laying (called pre-oviposition time) of *H. axyridis* was highly variable and negatively related to fecundity and egg viability. This study clarifies that the food readily available at adult eclosion does not affect the capacity of *H. axyridis* to reproduce, provided that adults find prey within a few days. It is concluded that the reproductive strategy of *H. axyridis* allows this ladybird beetle species to be highly adapted to heterogenous environments in its native range, highlighting the importance of diversifying agroecosystems to enhance biological regulation of pests.

**Abstract:**

While insect predators need high-quality food to reach sexual maturity and reproduce, starvation following adult eclosion may occur when prey are missing in agroecosystems. This study explores whether the type of diet available at eclosion determines the future fecundity of newly emerged adult predators. In a laboratory experiment, three different diets (i.e., flowers of *Perilla frutescens* (L.) Britton, eggs of *Ephestia kuehniella* Zeller as prey, or no food) were offered to adult females of the ladybird beetle *Harmonia axyridis* Pallas during their first three days after adult eclosion. On the fourth day, each female was paired with a prey-fed male and the pair was subsequently fed with prey. Diet at eclosion did not affect pre-oviposition time, the number of eggs oviposited daily, or the viability of egg batches. High variability in pre-oviposition time among females was observed for all diets. Significant negative linear relationships were found between pre-oviposition time and both the number of eggs oviposited daily and the viability of egg batches. This study clarifies that the food readily available at adult eclosion does not affect the capacity of *H. axyridis* to reproduce, provided that adults find prey within a few days. More generally, it shows that the reproductive traits of *H. axyridis* allow this generalist predator to be highly adapted to heterogenous environments in its native range. It is concluded that the variety of habitats offered by diversified agroecosystems may highly benefit the ladybird beetle *H. axyridis*, and potentially enhance its ability to biologically regulate crop pests.

## 1. Introduction

Insect predators play a crucial role in diversified agroecosystems by naturally regulating pests (e.g., [1]). Understanding their ecology is key to developing strategies that favor their presence and proliferation to facilitate biological control [2,3]. Various management practices can be applied in agricultural contexts to conserve and enhance predator populations in agroecosystems [4,5]. Notably, at the local scale, predators benefit from an increase in plant diversity [6] that can be enhanced by cultivating crop mixtures [7]. Semi-natural habitats rich in flowers can be created within fields or at field margins [8] to offer shelter, potential prey, and nectar and pollen resources [9]. Nectar and pollen can also be supplied by cultivating flowering crop species (potentially in association with a main crop), providing the double advantage of food and other consumables for people while benefiting flower-visiting predators [10].

Predatory ladybird beetles (Coleoptera: Coccinellidae) are important natural enemies of insect pests in agroecosystems [2,11]. Many species are well known as consumers of hemipteran pests such as aphids (Hemiptera: Aphididae) and some species also prey on young instars of other arthropods, notably Lepidoptera and Coleoptera [12,13]. In addition, they feed on pollen and floral nectar as well as on extrafloral nectar of plants [14], which partly explains their presence in flower-rich habitats [15]. While predatory ladybird beetles can potentially feed on a diversity of food sources, their fitness can be significantly affected by the quality of their diet (e.g., [16]). Hodek [17] distinguished essential food, which allows adults to oviposit and larvae to develop, from alternative food, which allows them to survive only. Mixed feeding on both essential and alternative food is common [18] and can enhance ladybird beetle fecundity [19], especially when essential food is limited [20]. Nonetheless, ladybird beetles may often experience starvation, a situation especially likely for just eclosed adults [21]. While females tend to optimize oviposition to the present and future availability of aphids, i.e., before the aphid density peak [22,23], their offspring may face prey scarcity already from the fourth instar larval stage [21]. Indeed, prey colonies may collapse due to the combined effect of predation and anti-predator response, loss of plant quality and plant defenses, i.e., by leaving the feeding site, dislodging from host plants, or producing winged morphs [24,25]. A continuous lack of availability of prey at larval and adult stages leads to slow larval growth, smaller size of surviving adults, longer pre-oviposition time, and reduced fecundity in ladybird beetles [26,27]. However, ladybird beetles often disperse at their adult stage, mainly by flying, to search for better food and reproduction sites [23,28]. Thus, it is likely that during their life, adults experience alternating periods of starvation and satiation in natural habitats.

*Harmonia axyridis* Pallas (Coleoptera: Coccinellidae) is a predatory ladybird beetle species native to East Asia (e.g., [29]). It has spread as an exotic species to many regions of the world, notably Europe and North and South America, mainly through deliberate introductions (e.g., [30]). In its native range, as well as in invaded areas, it is one of the most abundant ladybird beetles in agroecosystems, both in crop fields [31,32] and in semi-natural habitats rich in flowers [33]. It feeds on crop pests such as aphids [34], but it also consumes pollen and floral and extra-floral nectar [35,36]. The resource-tracking strategy of *H. axyridis* involves long- and short-distance movements through heterogeneous habitats with widely fluctuating food abundance, potentially of both prey and flowers, until ultimately finding a habitat favorable for oviposition [23]. Females respond to fluctuations in food supplies by switching between ovarian development and oosorption depending on prey availability [37]. However, they are not able to reproduce when feeding only on flowers [19].

Adult ladybird beetles freshly emerged from pupae in agroecosystems may have prey, i.e., crop pests or alternative prey, readily available; however, prey can often be missing in the habitats of beetle emergence. Alternatively, flowering plants managed by farmers, e.g., through the sowing of wildflower strips and/or the cultivation of flowering crops, may support predatory ladybird beetle populations by compensating for a lack of prey. In East-Asia and notably Japan, *Perilla frutescens* (L.) Britton (Lamiaceae) is an aromatic plant commonly grown in agroecosystems [38]. Previous research showed that *H. axyridis* females fed daily with five flowers of *P. frutescens* survived three-times longer than starving females [19]. From these results and their observations of females’ feeding behavior, Hatt and Osawa [19] suggested that *H. axyridis* females consume *P. frutescens*’ nectar and pollen when fed with flowers of this species (although it was not confirmed through further analyses, e.g., biochemical analyses of gut content). By using flowers of *P. frutescens*, the present study aims to clarify the critical importance of diet at adult eclosion by comparing the effects of a flower-based diet, prey food and starvation on the fecundity of *H. axyridis*. The link between diet, pre-oviposition time and fecundity, i.e., daily egg production and viability, is clarified and discussed in relation to potential applications for conservation biological control.

## 2. Materials and Methods

### 2.1. Experimental Design

Five males and five females of *H. axyridis* ladybird beetles collected at the botanical garden of Kyoto University (Kyoto, Japan; 35°03′ N, 135°79′ E) in June 2018 were paired and the pairs were individually reared to lay eggs in a plastic Petri dish (7 cm wide, 2 cm high). The produced larvae were grown up to the adult stage to check their sex ratio. One of the collected females laid only female progeny (six females only). Therefore, this female was removed from the original stock to eliminate the effect of male-killers for the following experiment. The remaining five males and four females were then placed in a plastic container (12 cm wide, 8 cm high). Several hundred adult offspring were obtained from these five males and four females, and their third-generation offspring was used for the experiment. From the parental generation (the field-collected individuals) to the larvae of the third generation, all the individuals reared in the laboratory were fed eggs of *Ephestia kuehniella* (Lepidoptera: Pyralidae) (Beneficial Insectary^®^, Guelph, ON, Canada) provided ad libitum.

Each pupa of the third generation was placed individually in a plastic Petri dish (7 cm wide, 2 cm high) with a filter paper and kept in an incubator (25 °C, 16:8 h light:dark photoperiod) until emergence. At emergence, the sex of each individual was determined. Males were then fed with *E. kuehniella* eggs. Females were randomly assigned to one of three diets: (1) five *P. frutescens* flowers provided daily (i.e., flowers); (2) *E. kuehniella* eggs provided daily (i.e., prey); and (3) no food (i.e., starving). Females were provided these diets over the first three days after eclosion. On the fourth day, each female was paired with a male aged more than three days, and the pair was then fed with *E. kuehniella* eggs until the end of the experiment.

In the present study, the different diets were provided to females over three days after eclosion because it was anticipated that the mortality of starving females would be high from the fourth day onwards [19]. Furthermore, *H. axyridis* may stay up to four days at the same habitat in fields before further dispersing [23]; therefore, the assumption was made in this study that all the beetles (i.e., including those which, at emergence, were starving and those which could feed on flower resources) could find prey from the fourth day after emergence.

Throughout the experiment, the ladybird beetles were offered their respective diet every day in a clean Petri dish with a filter paper. The quantity of *E. kuehniella* eggs provided daily corresponded to half a small laboratory spoon (0.027 ± 0.004 g, measured by weighing half a spoon of eggs, *n* = 20). Frozen *E. kuehniella* eggs were used as a substitute for natural prey, e.g., aphids. Although nutritional differences between *E. kuehniella* eggs and aphids, e.g., pea aphid *Acyrthosiphon pisum* Harris (Hemiptera: Aphididae), have been reported [39], *H. axyridis* can reproduce on a diet of *E. kuehniella* eggs with an egg batch size equivalent to that when feeding on *A. pisum* [40]. In addition, *E. kuehniella* eggs are intermediate suitable food for the development of *H. axyridis* [41]. Fresh flowers of *P. frutescens* were purchased on a regular basis during the experiment, always from the same provider (Syuyo, commercial supplier of *P. frutescens* flowers at Kyoto City central wholesale market, Kyoto, Japan). Flowers of *P. frutescens*, an ingredient in the local cuisine, can be purchased all year long. They were kept at 5 °C in the laboratory to always provide them fresh to the ladybird beetles. At the time of feeding the ladybird beetles, newly cut fresh flowers were introduced in each Petri dish hosting a female, and the flower remains of the previous day were removed.

If the male or the female of one pair died during the course of the experiment, the pair was excluded. Because of the number of ladybird beetles available from laboratory culture, the experimental trial was replicated twice. Each trial lasted for 90 days from eclosion. The first trial was conducted from February to May 2020, and the second from May to August 2020. The same generation of the ladybird beetles (i.e., the third generation) was used in both trials. More than 60 pairs were eventually used in each treatment, of which more than 40 pairs per treatment oviposited at the end of the two 90-day trials.

### 2.2. Data Collection

Each Petri dish was checked every day to assess whether the female was ovipositing. The pre-oviposition time of each female was calculated as the number of days between emergence and the first oviposition. Once the first oviposition was observed, the number of eggs laid was counted on a daily basis over 11 days. In the event of egg cannibalization by beetles, the remains of eaten eggs sticking to the filter paper or to the Petri dish were used to estimate the total number of oviposited eggs. Three egg batches per pair, usually the first three batches, were kept and placed in an incubator (25 °C and 16:8 h light:dark photoperiod) to assess their viability. Only batches with suitable eggs were kept; batches with too many damaged or cannibalized eggs were not considered. An egg batch was considered viable if at least one larva emerged from it. Egg hatchability (i.e., the rate of hatched larvae over the total number of eggs) was not considered here because frequent egg cannibalism by sibling larvae makes its accurate calculation uncertain.

### 2.3. Statistical Analyses

All statistical analyses were conducted using R software [42]. The Kaplan–Meier estimator was used to assess the effect of the diet (flowers, prey, or starving) offered to females over the first three days after eclosion on their pre-oviposition time (package ‘‘survival’’; [43]). The effect of diet was tested by using a log-rank test (package ‘‘survival’’; *p* < 0.05). The Kaplan–Meier method allows estimation of the proportion of females that never laid eggs, i.e., females at the pre-oviposition stage, at any given time of the experiment. It is especially appropriate here since some females never oviposited by the end of the experiment (i.e., “incomplete observations” also called “censored” data, [44]). To assess the effect of diet on the pre-oviposition time of females that oviposited (by excluding the censored data), a generalized linear model (GLM) was fitted with the negative binomial error distribution (log link function). This distribution was chosen to fix data overdispersion observed when the Poisson error distribution was used. Diet was included as a fixed factor and its effect was tested using a likelihood ratio test (*p* < 0.05).

The effect of diet on the number of eggs laid was assessed by fitting a generalized linear mixed effect model (GLMM, package ‘‘lme4”; [45]) with the negative binomial error distribution (log link function). Diet was included as a fixed factor, and ladybird beetle pairs (identified by a single number) nested within their respective trial were included as a random factor because eggs laid by the same pairs were counted over 11 successive days. The effect of diet was tested using a likelihood ratio test (*p* < 0.05).

The effect of pre-oviposition time (of the females that oviposited) on the number of eggs laid was assessed through linear regressions (*p* < 0.05). The mean number of eggs laid per female over 11 days was calculated and log_10_(x + 1) transformed prior to the analysis. Linear regressions were conducted for each diet separately.

The effect of pre-oviposition time and diet on the rate of viable batches (batches with at least one hatched egg) was assessed by fitting GLMs with a binomial error distribution (logit link function). A binomial distribution was used since hatching was considered a binary response, i.e., at least one egg hatched within the batch vs. no egg hatched. Pre-oviposition time, diet and their interaction were included as fixed factors and their effects were tested using a likelihood ratio test (*p* < 0.05). As three batches were collected per pair of ladybird beetles, three levels of viable batches were described for each pair: (1) at least one batch had an egg hatched; (2) at least two batches had an egg hatched; (3) all three batches had an egg hatched. A GLM was fitted for each of the three categories. When less than three batches could be collected for a given pair, because less than three batches were oviposited or because eggs were too damaged or cannibalized, the missing egg batches were considered as batches with no egg hatched.

## 3. Results

### 3.1. Effect of Diet at Adult Eclosion

**Figure 1:**
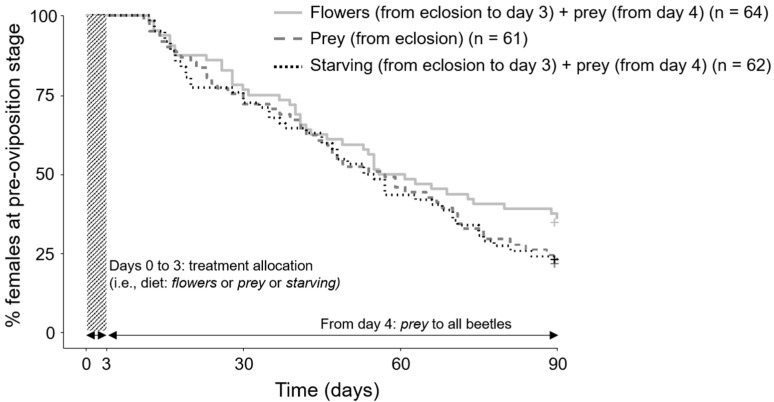
Kaplan–Meier curves showing the percentage of *Harmonia axyridis* females at pre-oviposition stage through time according to their diet (i.e., flowers, prey, or starving; diet offered over the first three days after eclosion, then all fed with *Ephestia kuehniella* eggs) plotted using the R package ‘‘survival’’ [43]. All the tested females were included in the analysis (including those that did not lay eggs at 90 days after emergence).

The type of diet offered to *H. axyridis* females over the first three days after emergence did not significantly affect their pre-oviposition time when considering all the tested females (i.e., including both those that oviposited within 90 days after emergence and those that did not; *df* = 2; *χ*^2^ = 2.2; *p* = 0.3; Figure 1). In the case of the ladybird females that oviposited within 90 days after emergence (flowers: 41 out of 64; prey: 47 out of 61; starving: 47 out of 62), the type of diet also did not significantly affect their pre-oviposition time (*df* = 2; *χ*^2^ = 0.266; *p* = 0.875). Their pre-oviposition time was 42.7 ± 21.7 days (mean ± SE) with a median value of 41 days for those fed with flowers, 45.1 ± 23.5 days with a median value of 44 days for those fed with prey, and 43.2 ± 22.5 days with a median value of 45 days for those starving.

The type of diet offered over the first three days after emergence did not significantly affect the daily number of eggs laid by *H. axyridis* females (*df* = 2; *χ*^2^ = 0.164; *p* = 0.921). The daily fecundity was 23.7 ± 20.6 eggs for those fed with flowers, 22.7 ± 19.0 eggs for those fed with prey, and 22.9 ± 19.7 eggs for those starving.

Finally, the type of diet offered over the first three days after emergence did not significantly affect the rate of viable batches (Table 1 and Table 2).

### 3.2. Effect of Pre-Oviposition Time

**Figure 2:**
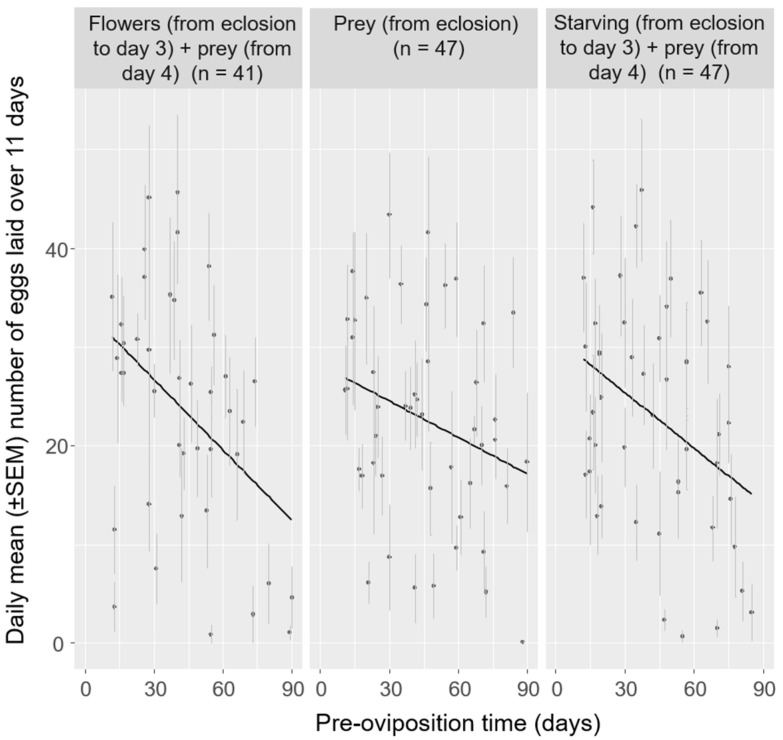
Linear regression of pre-oviposition time and the daily mean number of eggs laid per *Harmonia axyridis* female over 11 days for each diet (i.e., flowers, prey, and starving) plotted using the R package “ggplot2” [46]. See Table 3 for statistical results of the analysis.

The variability in pre-oviposition time was large for females provided with the same diet (Figure 2). Significant negative linear relationships were found between pre-oviposition time and the mean number of eggs laid per day: *H. axyridis* females with shorter pre-oviposition time laid significantly more eggs every day on average, and the same tendency was observed for all diets (Figure 2; Table 3). Moreover, significant negative linear relationships were found between pre-oviposition time and the rate of batches with hatched eggs: *H. axyridis* females with shorter pre-oviposition time laid more egg batches that were viable (Table 2).

## 4. Discussion

### 4.1. Effect of Diet at Adult Eclosion on Pre-Oviposition Time and Fecundity

The results of this study indicate that the diet (flowers, prey, or starving) of the ladybird beetle *H. axyridis* at emergence does not significantly affect its reproductive traits: pre-oviposition time (Figure 1), the number of eggs oviposited daily, or the viability of egg batches (Table 1 and Table 2). These results partly agree with those of Wolf et al. [36] who reported that the number of eggs produced by freshly emerged *H. axyridis* females fed with flowers over six days after eclosion was comparable to the number produced by starving females; however, they also found that females fed with flowers had a shorter pre-oviposition time (less than 15 days on average for flower-fed females compared to about 16 days on average for starving females), which was not the case in our study. Wolf et al. [36] did not consider prey-fed females in their study. In addition, Dixon and Agarwala [47] reported an extended pre-oviposition time in *H. axyridis* females fed with honey (potentially simulating nectar) for five days after eclosion (about 14 days on average) compared to aphid-fed females (about eight days on average). Compared to the present study, Dixon and Agarwala [47] included neither fresh flowers nor starvation as treatments. To the best of our knowledge, the present research is the first to assess the importance of prey food, flower food, or an absence of food, available over the first days after adult emergence, on the future fecundity of a predaceous ladybird beetle.

The length of the pre-oviposition period of *H. axyridis* females can explain the lack of effect of diet at eclosion on their fecundity (i.e., the daily number of eggs laid and egg batch viability). The first females to lay eggs did so more than 10 days after eclosion (Figure 2), and most of the tested females oviposited for the first time more than six weeks after emergence. *Harmonia axyridis* females have no eggs in their gonads at eclosion [47]. These last authors suggested that eggs start to develop in the ovaries from the very first days after emergence when females are fed with prey. They also observed that postponing prey availability to newly emerged females delayed their first oviposition in a population of *H. axyridis* displaying a pre-oviposition time of less than 10 days on average. This contrasts with the results of this study as females laid their first eggs more than six weeks after emergence on average. It can be hypothesized that when the pre-oviposition time increases, the potential effect of diet at eclosion is diluted. It appears that the availability of prey from the fourth day after emergence allowed an egg development that was comparable between all females independently of their diet at eclosion.

High variability in pre-oviposition time was observed for all of the tested diets (Figure 2). Theoretical studies have clearly shown that a short pre-oviposition time (i.e., an early start to oviposition) generally increases fitness in many organisms [48,49,50], which may result in little variation in pre-oviposition time between individuals. Less attention has been paid to cases when individual variation in pre-oviposition time is important. In this study, high variability was observed in the pre-oviposition time of *H. axyridis*, although all females were fed with prey from the fourth day. This high variability in the pre-oviposition time independent of diet indicates that favorable dietary conditions just after emergence (i.e., the availability of prey, compared to flower food or starvation during the first three days from eclosion) do not necessarily trigger oviposition. It represents a potential advantage in relation to the fluctuation of prey availability in natural environments. In Kyoto (in central Japan, within the native range of *H. axyridis*) for example, the abundance of several species of aphid generally peaks first at the end of April, second in mid-June [23,51], and third in October (personal observation). These multiple peaks of good-quality prey represent different windows for oviposition from spring to autumn. The intrinsic variability of pre-oviposition time among females makes possible a spreading through time of oviposition across the different windows in heterogenous environments and partially limits intra-specific competition for available resources [21,23].

The high variability in pre-oviposition time, potentially leading to an extended period from emergence to the first oviposition, may increase the chances of females finding high-quality food (i.e., prey) before ovipositing (although it may be at the cost of encountering more factors contributing to mortality). While a short period of food deprivation may be a common condition of freshly emerged adults [21], it does not necessarily endanger the capacity of *H. axyridis* individuals to reproduce, provided that they find prey within a few days.

### 4.2. Negative Relationships between Pre-Oviposition Time and Fecundity

In this study, negative relationships were observed between pre-oviposition time and fecundity (i.e., the daily number of eggs laid and egg batch viability) of *H. axyridis*, independent of the type of diet (Figure 2; Table 2 and Table 3). Such relationships may not have been directly reported previously, but they resemble previous findings on other ladybird beetle species showing negative relationships between (i) longevity and daily fecundity [47], and (ii) late mating and egg batch viability [52]. Indeed, late mating, prolonged pre-oviposition time, and aging may be potentially correlated. These negative relationships are consistent with the generally accepted assumption that there is a clear advantage in early reproduction as the probability of being able to reproduce is greater early than late in adult life [2]. However, this study shows that not every individual in a native population reproduces shortly after emergence. While late egg laying is disadvantageous, as it is related to reduced fecundity, late oviposition may have some potential benefits. Two hypotheses are proposed. Firstly, laying fewer eggs and laying late may be part of a survivorship strategy. *Harmonia axyridis* females that produce fewer eggs tend to live longer [26]. While a link between pre-oviposition time and longevity has not been reported in ladybird beetles, it has been shown in other animals that females that reproduce late live longer (e.g., [53]). Nonetheless, in the ladybird beetle *Propylea dissecta* (Mulsant) (Coleoptera: Coccinellidae), late-mating individuals were shown to lay a reduced number of eggs with lower viability [52], suggesting that laying eggs late, consequently, laying fewer eggs, was part of some females’ strategy to live longer. An extended life span can allow spring-born *H. axyridis* females to live until autumn, overwinter, and oviposit only in the next spring to produce a new generation. Secondly, late egg laying can be part of a risk-spreading strategy, i.e., bet-hedging [54]. Typically occurring in natural insect populations, risk spreading consists of the development of genotypes with lower variance in fitness at the cost of lower mean fitness of populations facing unpredictably variable environments [55]. *Harmonia axyridis* females, even when encountering good conditions for oviposition, e.g., an abundance of prey, may not lay eggs, but disperse further to finally distribute their eggs widely among prey patches, thus insuring against potentially random collapse of favorable conditions [56]. While past research considered delaying oviposition as a risk-spreading strategy for potentially sexually mature insect females (e.g., [57,58]), this strategy may also apply to females that have never laid eggs by delaying their first oviposition.

### 4.3. Contrasts between Native Populations and Exotic and Long-Term Laboratory Populations of Harmonia axyridis

The findings of this study reveal the reproductive strategy of a native population of *H. axyridis* which was reared in the laboratory until the third generation (F3) only. They are in contrast to the findings for exotic and long-term laboratory populations. Notably, exotic populations of *H. axyridis* generally display a shorter pre-oviposition period: for example, 20 to 30 days in field populations and about 10 days in a long-term laboratory population, i.e., the 50th generation, in Belgium (fed with *E. kuehniella* at 23 ± 1 °C and 16:8 h light:day), with little variation between individuals [40]. Outside its natural range, *H. axyridis* was intentionally introduced as a biological control agent after mass rearing in several countries before it spread further into neighboring regions [59,60]. It appears that mass rearing of *H. axyridis* for biocontrol purposes selects individuals displaying strategies for rapid reproduction, notably a short pre-oviposition time and egg production concentrated in the few days after eclosion [61]. Similarly, studies using long-term stock cultures of *H. axyridis* show that ladybird beetles harbor reproductive traits such as a short pre-oviposition time (i.e., 10 to 15 days) with little variability between individuals, high daily fecundity (i.e., 35 to 40 eggs per day), and egg laying over a limited period (e.g., [36,40,47]). Hence, the large variation in pre-oviposition time observed in this study may reflect one of the distinct characteristics of *H. axyridis* individuals in natural populations adapted to heterogenous environments (but see also [26]).

### 4.4. Implications for Conservation Biological Control

As the diet at eclosion does not affect the future fecundity of *H. axyridis*, the results of this study suggest that flower food in agroecosystems is not critically important for freshly emerged adults to compensate for a lack of prey, provided that female adults find prey from the fourth day onward after emergence. Nonetheless, the availability of floral resources remains important in situations of prey scarcity for ladybird beetles to survive. Indeed, it could be hypothesized that female beetles cannot find prey during more than three days after emergence and previous research showed that 75% of *H. axyridis* females in a population do not survive starving for more than four days from emergence [19]. Future research could test the effect of a prolonged flower-based diet (i.e., longer than three days from eclosion) on females’ future fecundity. While scarcity of common prey (e.g., aphids) may also favor cannibalism and intraguild predation by adult beetles [62], potentially disrupting biological control [63], future research could also test whether flower food available at adult eclosion diverts adult *H. axyridis* from preying on their conspecifics and other intraguild predators.

The present results suggest that *H. axyridis* would benefit from spatially diversified agroecosystems made of various habitats within which different prey species occur at different times [64,65], creating continuity in food resources for predators [66], and offering many opportunities for them to find prey and reproduce while potentially avoiding intra- and inter-specific competition [3]. As *H. axyridis* may exploit the various food resources available through time and space, its predation efficiency is expected to be high [67]. Hence, the reproductive behavior of *H. axyridis* in this study, along with its resource-tracking strategy previously studied in its native range [23], suggests that diversification of agroecosystems is promising for conserving natural populations of this insect predator. By better understanding predator behavior and reproductive strategies in relation to food availability, this study can help develop agroecosystem designs allowing the conservation of natural enemies of pests.

## Figures and Tables

**Table 1:** Number of *Harmonia axyridis* females producing viable and not viable egg batches (i.e., Yes: hatched egg batch; No: not hatched egg batch), and rate of females producing viable egg batches over the total number of ovipositing females (in percentage), according to the diet they received at eclosion. A maximum of three batches were collected from each female.

	No	Yes	Rate (%)
**At least one batch hatched**	**11**	**124**	**91.9**
Flower	4	37	90.2
Prey	2	45	95.7
Starving	5	42	89.4
**At least two batches hatched**	**20**	**115**	**85.2**
Flower	9	32	78.0
Prey	4	43	91.5
Starving	7	40	85.1
**All three batches hatched**	**45**	**90**	**66.7**
Flower	14	27	65.9
Prey	18	29	61.7
Starving	13	34	72.3

**Table 2:** Effect of pre-oviposition time, diet (flowers, prey, or starving), and their interaction on the rate of viable egg batches (degree of freedom (*df*), chi-square value (*χ*^2^) and *p*-value (*p*) from the likelihood-test for generalized linear models (GLM) are provided, *** *p* < 0.001).

	*df*	*χ* ^2^	*p*	Estimate ^1^
**At least one batch had hatched eggs**				
Pre-oviposition time	1	12.75	<0.001 ***	-
Diet	2	2.503	0.286	
Pre-oviposition time × diet	2	1.884	0.39	
**At least two batches had hatched eggs**				
Pre-oviposition time	1	16.79	<0.001 ***	-
Diet	2	4.766	0.092	
Pre-oviposition time × diet	2	4.503	0.105	
**All three batches had hatched eggs**				
Pre-oviposition time	1	13.59	<0.001 ***	-
Diet	2	1.117	0.572	
Pre-oviposition time × diet	2	0.126	0.939	

^1^ Sign of estimate (i.e., factor’s coefficient) obtained from the fitted GLM is provided when a factor shows a significant effect on the tested variable. A negative estimate indicates that the factor has a negative effect on the tested variable.

**Table 3:** Effect of pre-oviposition time on the daily mean number of eggs laid per female over 11 days for each diet (linear regression with log_10_(x + 1)-transformation prior to analysis, *: *p* < 0.05; ** *p* < 0.01). (See also Figure 2).

	*df*	*F*	*p*	*R* ^2^
Flowers	1, 39	9.391	0.004 **	0.19
Prey	1, 45	4.845	0.033 *	0.10
Starving	1, 45	8.022	0.007 **	0.15
